# Supplementary material for: The role of orthographic neighbourhood effects in lateralized lexical decision: a replication study and meta-analysis
Source: PeerJ. 2021 Apr 28;9:e11266. doi: 10.7717/peerj.11266 (PMC8088209; doi:10.7717/peerj.11266)
Supplement: Supplemental Information 1 — (A) N effects across visual fields for each participant; (B) Inclusion criteria for the meta-analysis; (C) Visualisation of publication bias; (D) AXIS scoring; (E) Sensitivity Analysis [file peerj-09-11266-s001.docx]

**Supplemental Materials**

**The role of orthographic neighbourhood effects in lateralized lexical decision:**

**A replication study and meta-analysis.**

**Parker, A. J., Egan, C., Grant, J. H., Harte, S., Hudson, B. T., and Woodhead, Z. V. J.**

**Supplemental Materials A: N effects across visual fields for each participant**

Table S1. Individual reaction time differences (ms) for the high N - low N stimuli across each visual field. A negative value indicates a facilitatory effect and a positive value indicates an inhibitory effect.

|  | Visual field | | |
| --- | --- | --- | --- |
| Participant | Left | Central | Right |
| 1 | 27.2 | 19.0 | 8.9 |
| 2 | -11.8 | -63.2 | 78.9 |
| 3 | -19.7 | -33.9 | 76.2 |
| 4 | -24.7 | 30.9 | 77.1 |
| 5 | -162.8 | -66.0 | -102.8 |
| 6 | -7.8 | -41.1 | 25.3 |
| 7 | 30.1 | 12.2 | 36.4 |
| 8 | 86.5 | -7.2 | -62.0 |
| 9 | -93.5 | 80.3 | -41.7 |
| 10 | 44.8 | 88.0 | 119.6 |
| 11 | 3.2 | 4.4 | 21.1 |
| 12 | -55.3 | -8.9 | 13.2 |
| 13 | 3.8 | 20.3 | -60.4 |
| 14 | -6.4 | -66.8 | 35.3 |
| 15 | -105.7 | -30.4 | 56.1 |
| 16 | -23.2 | 10.0 | -36.2 |
| 17 | 139.3 | -98.8 | 124.8 |
| 18 | -72.5 | -33.1 | 10.7 |
| 19 | -13.8 | 10.1 | 7.6 |
| 20 | 9.3 | 17.0 | 10.8 |
| 21 | -31.1 | 54.6 | 7.3 |
| 22 | 98.7 | 56.6 | 2.1 |
| 23 | -6.6 | 15.2 | 103.9 |
| 24 | -132.5 | -13.6 | 106.3 |
| 25 | -114.0 | 18.9 | -19.8 |
| 26 | -36.2 | -29.9 | -55.6 |
| 27 | -135.6 | -4.0 | -27.9 |
| 28 | 4.3 | 10.1 | -64.6 |
| 29 | -70.6 | -37.0 | -17.6 |
| 30 | -1.2 | 32.2 | -35.1 |

**Supplemental Materials B: Inclusion criteria for the meta-analysis:**

- The study investigated the effect of experimentally manipulated orthographic neighbourhood size during a lateralized lexical decision task. Manipulations of N and visual field must be within subjects and the visual field manipulation must include at least left and right visual field presentation.*
- The study reported lexical decision reaction times.
- The study did not involve priming.
- The study used an alphabetic language that is read from left to right.
- Participants were native readers of the language used for the lexical decision task.
- People with history language impairment or dyslexia, or any neurological condition such as epilepsy were excluded. If there is no explicit mention of these factors, then it will be assumed they did not apply.*
- Participants in the studies were aged between 18 and 50 years old.

* Note. Studies which include additional manipulations will be included. We will aggregate data across these conditions so that we have cell means for the 2 (Nsize) by 2 (visual field)

manipulation, so long as the manipulation was within item.

** If studies include control groups (i.e. those without language impairment or dyslexia), we will include data for the control group in our analysis.

**Supplemental Materials C: Visualisation of publication bias**


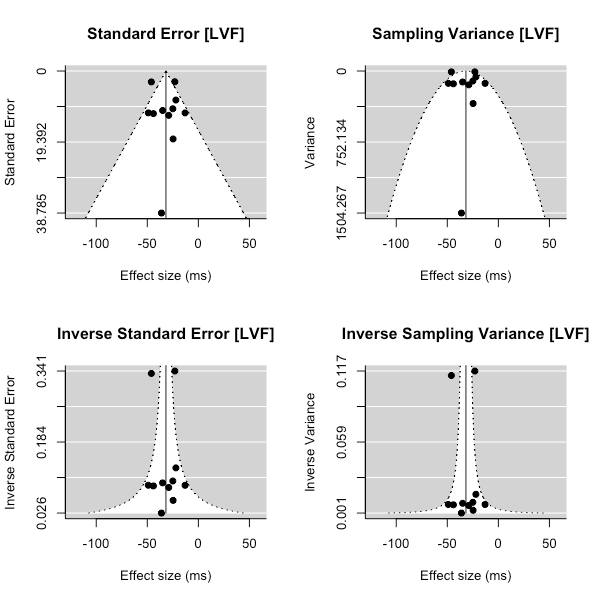


Figure S1. Funnel plots of the studies included in the meta-analysis for the LVF. In the absence of bias, more precise studies are expected to appear narrowly at the top of the plot, whereas less precise studies are expected to scatter more widely at the bottom of the plot.


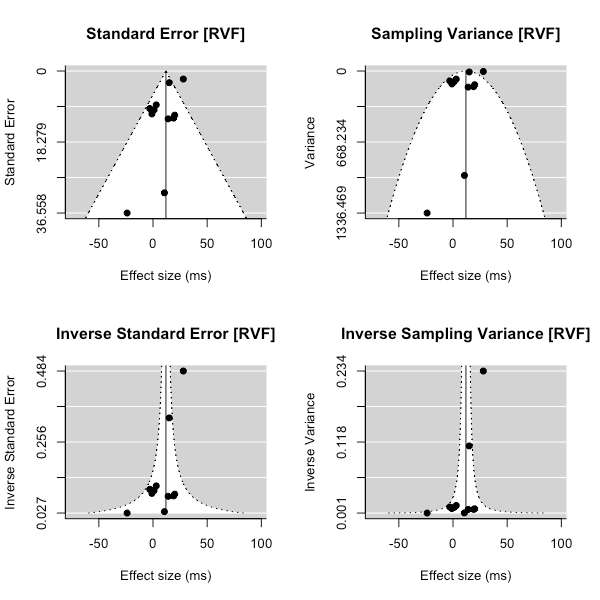


Figure S2. Funnel plots of the studies included in the meta-analysis for the RVF. In the absence of bias, more precise studies are expected to appear narrowly at the top of the plot, whereas less precise studies are expected to scatter more widely at the bottom of the plot.

**Supplemental Materials D: AXIS scoring**

Table S2. AXIS score for each article included in the meta-analysis.

|  | AXIS score | |
| --- | --- | --- |
| Article | Reviewer 1 | Reviewer 2 |
| Lavidor & Ellis (2001) | 10 | 10 |
| Lavidor & Ellis (2002) | 9 | 9 |
| Lavidor et al. (2006) | 6 | 7 |
| Perea et al. (2008) | 9 | 8 |
| Mano et al. (2010) | 11 | 11 |
| Whitney & Lavidor (2005) | 8 | 8 |

*Note*. Scores could range between 0 and 15.

**Supplemental Materials E: Sensitivity Analysis**

None of the reviewed studies reported the correlation coefficient for the relationship between reaction times in the high- and low-N conditions in any visual field. Thus, we calculated the variance associated with the observed effect in each study by assuming that the correlation coefficient would be identical to that observed in our replication study. To examine the influence of this estimate on our conclusions, we conducted sensitivity analysis where we repeated the meta-analysis under the assumption that *r*= .2, .5, or .8. The outcome of these meta-analyses were consistent; the N effect was always facilitatory in the LVF and always inhibitory in the RVF, irrespective of the size of the correlation coefficient used to calculate the variance of each study. The estimates produced when using variable *r* values are shown in Table S3 along with their corresponding *p*-values.

Table S3. Estimates (*ms*) of the N effect in the left visual field (LVF) and right visual field (RVF) when varying the correlation coefficient (*r*) used to obtain variances and effect sizes in the meta-analysis.

|  | *r*= .2 | | *r*= .5 | | *r*= .8 | |
| --- | --- | --- | --- | --- | --- | --- |
|  | estimate | *p*-value | estimate | *p*-value | estimate | *p*-value |
| LVF | -32.7 | < .001 | -32.4 | < .001 | -31.7 | < .001 |
| RVF | 16.2 | .001 | 14.6 | .002 | 11.9 | .008 |
